# Plant-Based Beverages: Consumption Habits, Perception and Knowledge on a Sample of Portuguese Citizens

**DOI:** 10.3390/foods13203235

**Published:** 2024-10-11

**Authors:** Ofélia Anjos, Patrícia C. P. Pires, Joana Gonçalves, Letícia M. Estevinho, António G. Mendonça, Raquel P. F. Guiné

**Affiliations:** 1 CERNAS-IPCB—Centro de Investigação em Recursos Naturais, Ambiente e Sociedade, Instituto Politécnico de Castelo Branco, 6001-909 Castelo Branco, Portugal; 2 CEF, Forest Research Centre, Associate Laboratory TERRA, School of Agriculture, University of Lisbon, 1349-017 Lisboa, Portugal; 3 Spectroscopy and Chromatography Laboratory, CCBP-BI, Centro de Biotecnologia de Plantas da Beira Interior, 6001-909 Castelo Branco, Portugal; 4 CICS–UBI–Health Sciences Research Centre and Chemistry Department, University of Beira Interior, 6200-506 Covilhã, Portugal; patricia.carmonapires@gmail.com (P.C.P.P.); mendonca@ubi.pt (A.G.M.); 5 CERNAS-IPV Research Centre, Polytechnic University of Viseu, 3504-510 Viseu, Portugal; joanadgoncalves13@gmail.com; 6 Mountain Research Center (CIMO), Polytechnic Institute of Bragança, 5301-854 Bragança, Portugal; leticia@ipb.pt; 7 Laboratorio para a Sustentabilidade e Tecnologia em Regiões de Montanha, Instituto Politécnico de Bragança, Campus de Santa Apolónia, 5300-253 Bragança, Portugal

**Keywords:** plant-based beverages, questionnaire survey, almond, soy, oat

## Abstract

Plant-based beverages (PBB) consumption has increased significantly worldwide due to an interest in vegetarian/vegan diets, taste preferences, health and ethical and environmental issues. Therefore, this study intends to investigate consumption habits, consumer preferences and consumers’ level of knowledge about PBB. In this study, a voluntary, anonymous questionnaire survey was applied to a sample of participants from Portugal. The sample was recruited by convenience, and therefore, the distribution among the groups was not even. Data analysis involved different statistical techniques: basic statistics, chi-square tests, factor analysis, cluster analysis and tree classification analysis. The results indicated that the most consumed PBB were almond, soy and oat beverages. The majority of consumers chose these beverages for nutritional and health reasons, while a smaller number consumed them as part of a vegetarian or vegan diet. The main motivations for consuming PBB are mainly associated with sustainability and health benefits. The results regarding the respondents’ knowledge about PBB revealed that a health-related profession was the most significant predictor. These results allowed us to conclude that the factors of nutrition, health, ethics and practice of a vegetarian/vegan diet influence the consumption of PBB. It was also concluded that being in a healthcare profession, along with age and professional status within this field, were significant factors influencing the level of knowledge about PBB.

## 1. Introduction

Plant-based beverages (PBB) consist of water-soluble extracts of legumes, namely the following: (i) soybean (*Glycine max*) and chickpea (*Cicer arietinum*); (ii) seeds like sunflower (*Helianthus annus*) and sesame (*Sesamum indicum*); nuts, such as almond (*Prunus dulcis*), cashew nut (*Anacardium occidentale*), hazelnut (*Corylus avellana*) and Brazil nut (*Bertholletia excelsa*); (iii) cereals such as rice (*Oryza* spp.) and oats (*Avena sativa*) or (iv) pseudocereals quinoa (*Chenopodium quinoa*) [1,2,3]. The consumption of PBB is growing significantly worldwide (about 10.18% each year between 2020 and 2024), owing to interest in diets free from animal products, ethical and environmental concerns and taste preferences [4,5]. Another reason associated with this increase is also because some consumers are lactose intolerant (65.0 to 75.0%) or allergic to milk (0.5 to 3.5%) [4]. This interest in “healthier” foods has led the food industry to develop new products whose functions are intended to go beyond the supply of primary nutrients and food satisfaction [6].

Consumers look for a product with organoleptic characteristics and sensory aspects similar to milk [7]. However, when it comes to nutritional aspects, there are a few similarities between PBB and milk [4]. Despite this, PBB provide nutrients and bioactive substances that contribute to health improvement, positively impacting obesity, hypertension, type 2 diabetes, cardiovascular diseases, metabolic syndrome and cancer [8,9]. Vanga and Raghavan [8] conducted a review on the nutritional composition of PBB as compared to cow’s milk, used as a reference, and they highlighted some pros and cons of some PBB, namely a soy beverage, a almond beverage, a coconut beverage and a rice beverage, concluding that soy and almond beverages were the best alternatives to cow milk, considering a balanced diet, the presence of proteins and low calories. Additionally, these beverages contain much higher quantities of unsaturated fatty acids, proteins, dietary minerals and vitamins than cow milk. The review by Sethi et al. [9] highlights the role of PBB as functional beverages because they contain functionally active components that bring health-promoting properties. These components include phenolic compounds, isoflavones, phytosterols, or vitamin E, and their established health benefits include protective effects against cancer, cardiovascular diseases, osteoporosis, high cholesterol, hypertension or ageing. Also, they have anticarcinogenic, antitumor and antiviral activities, and promote brain health [9]. Although the nutritional composition of these beverages is variable, the presence of isoflavones and phytosterols (soy beverage), α-tocopherol and arabinose (almond beverage) and β-glucan (oat beverage) can be highlighted [4,10].

To understand and evaluate the opinions of buyers and consumers and to realise their core beliefs and PBB consumption patterns, market research studies were performed [11,12,13]. A clear understanding of consumers’ attitudes is essential to improve the existing knowledge and to develop new PBB [14]. The study of consumer behaviour has a multidisciplinary character, involving several areas such as food science, health and technology, nutrition, psychology and marketing [6,15]. Consumers’ perception of the characteristics of a product can be influenced by several individual factors that affect the perception of sensory attributes, which interact with physiological, behavioural and cognitive factors [16]. The taste acceptance of current or proposed PBB need to be determined by experiment (Decker 2004). This acceptance depends on the consumer’s consideration of nutrient content and its taste [17,18], but also on the consumers’ attitude—a predisposition developed by the individual, formed from their experiences and information obtained [6]. Consumers’ attitude is a behavioural process that can shed light on the reasons why consumers will adopt or decline the use of PBB and are strongly influenced by personal experiences with the product during product usage. Also, expectation plays an important role in relation to the consumption of food products because it can benefit or impair the perception of the product by the consumers [19,20]. As the attributes associated with that characteristic are already evaluated or given positive or negative values, consumers simultaneously and automatically acquire an attitude towards this product [21]. Consumer beliefs and subsequent attitudes towards a product influence the acceptability of that product. The work by Eccles and Wigfield [21] describes a review of motivational beliefs, values and goals. It highlights that individuals can be intrinsically or extrinsically motivated towards certain activities or products. Based on these principles, when consumers are intrinsically motivated, they accept the product because they are interested in it and enjoy it. On the other hand, when consumers are extrinsically motivated, they accept it for other reasons, such as benefits/rewards. As such, with the knowledge about possible beneficial nutritional and health effects of PBB, this “reward” acts as a trigger for an automatic positive attitude and acceptance.

To date, few studies have explored consumers’ beliefs, perceptions and acceptance towards PBB [22,23,24]. A variety of studies have assessed the effects of different factors on the sensory characterisation and acceptance of products [25]. However, there is a lack of published literature dealing with ethnic differences in sensory evaluation and acceptance of foods [15,26], particularly regarding the consumption of PBB as an alternative to animal milk. In this matter, the attitudes, preferences and knowledge of Portuguese consumers regarding plant-based alternatives to milk from animal origin have not yet been investigated.

The process of developing a new product frequently takes place due to the existence of a demand or the explicit need for its consumption. The interest in investigating this topic can be explained, in part, by the changes in attitudes, beliefs, values and motivations of these consumers who have taken a critical stance in relation to the consumption of industrialised products [27]. The success of the food manufacturing industry is based on knowing consumers by recognising and understanding the beliefs that influence attitude formation, which affects their decisions about a product [28].

Consumer surveys and market studies are key factors for the success of these newly developed products and allow us to foresee the positive and less positive aspects valued by the potential future buyers in time to implement corrections if that would be the case [29]. The scientific literature is very scarce about studies undertaken on consumers about their habits and knowledge related to PBB. There are some studies that focus on the acceptance of PBB from the sensorial point of view, which is a challenge given that their taste is highly different from animal milk, such as the studies by Amyoony et al. [30] or Jaeger et al. [31]. However, the motivators for the consumption and level of knowledge about these beverages are under-investigated. Therefore, the aim of the present work was to explore consumers’ beliefs and to investigate the consumer’s acceptance towards new PBB with functional ingredients prior to their commercialisation. The developed beverages would have health benefits owing to the incorporation of different ingredients with diverse biological effects, and the consumers would express their possible degree of approval and buying intentions by answering a questionnaire [14,32]. Also, we aimed to evaluate to what extent the socio-demographic characteristics of a sample of the Portuguese population influence the knowledge and consuming habits related to PBB.

## 2. Materials and Methods

### 2.1. Instrument

A questionnaire was created to undertake a market study aimed at investigating the potential for marketing a new type of PBB and evaluating consumer acceptance. The questionnaire included four sections designed to collect information on several topics:

- Part I—Socio-demographic data (1. Age class, 2. Gender, 3. Education Level, 4. Civil State, 5. Profession, 6. Professional activity related to one of the areas: Nutrition, Agriculture, Health, Other, 7. Residence Area);
- Part II—Consumption habits related to plant-based drinks (1. Do you consume PBB?, 2. What PBB do you prefer?, 3. How often do you consume these types of beverages?, 4. What are the reasons for your choice?;
- Part III—Acceptance of the new product (1. What are the reasons for non-consumption?, 2. What would be your acceptability to consume drinks obtained from plants?, 3. What would be your acceptability to eat dishes made with these drinks?, 4. What reasons could contribute to making you consume PBB?;
- Part IV—Perceptions and Knowledge about PBB. (1. Perceptions and beliefs towards PBB, 2. Do you agree that it is necessary to develop a new product with an appealing flavour?). Regarding the first part about perceptions and knowledge, 23 items were used and the respondents had to express their agreement towards each item on a Likert scale of I points, from 1 = totally disagree to 5 = totally agree, with a score 3 = neither agree nor disagree. The items were as follows:
  1. PBB have less nutritional value than milk;
  2. PBB are difficult to digest;
  3. PBB have a greater risk of pesticide residues than milk and dairy;
  4. PBB are expensive;
  5. PBB have an unpleasant colour and taste;
  6. There are a wide variety of PBB on the market;
  7. The consumption of PBB helps alleviate the symptoms of menopause;
  8. Replacing milk with PBB contributes to the reduction in body fat;
  9. The consumption of PBB helps to strengthen bones;
  10. Herbal drinks have a lower risk of antibiotics than milk and milk products;
  11. The consumption of PBB helps to reduce cholesterol;
  12. Vegetable drinks are an option for lactose intolerants;
  13. In developed countries, PBB are not consumed;
  14. PBB are part of Western culture;
  15. PBB are characteristic of developed counties;
  16. PBB are a source of energy;
  17. PBB are rich in macro and micronutrients;
  18. PBB contain vitamins;
  19. PBB contain dietary minerals such as calcium, iron and magnesium;
  20. PBB contain bioactive compounds with health benefits;
  21. PBB are a source of vitamin D;
  22. PBB have antioxidant properties;
  23. PBB have anti-inflammatory properties.

Note that the questionnaire was applied in the Portuguese language, and no abbreviations were used in the questions for the participants. Although the abbreviation PBB is used in the article for simplicity, the original words used were “Bebidas Vegetais”.

The data collection took place in Portugal between February and March 2021; convenience sampling was performed. The questionnaire was sent to a high number of people and institutions, including companies and universities/faculties, selected according to some personal relations and privileged professional contacts. Additionally, it was requested that they announce the survey to their personnel via institutional e-mail as a way to obtain to a broader audience and have more success in accepting participants in the study and replying to the questionnaire.

All data collected were treated with confidentiality and met all ethical issues so that it was impossible to link the answers to a particular individual. From the 903 responses obtained, 17 were deemed invalid because the participants did not explicitly express their consent to participate in the survey. Hence, a total of 886 valid responses were collected from different Portuguese regions. Only individuals ≥18 years old were eligible, and all ethical issues were followed when designing and applying the questionnaire, which was applied only after informed consent. Study procedures and documents were approved by the Ethical Committee at the University of Beira Interior (code No. CE-UBI-Pj-2020-098).

### 2.2. Data Analysis

Socio-demographic information was collected and age was classified into categories as follows: young adults (aged between 18 and 30 years), middle-aged adults (between 31 and 50 years), senior adults (between 51 and 65 years) and the elderly (aged 66 years or over). For data analysis, different basic descriptive statistical tools were used, such as frequencies and descriptives, including minimum, maximum, mean value and standard deviation. The crosstabs and the chi-square test were used to assess the associations between some of the categorical variables under study. Moreover, the Cramer’s V coefficient was used to analyse the strength of the significant relations found between some of the variables. This coefficient ranges from 0 to 1 and can be interpreted as follows: V ≈ 0.1, the association is considered weak; V ≈ 0.3, the association is moderate; and V ≈ 0.5 or over, the association is strong. The variables accounting for the average level of knowledge of the categories of PBB were submitted to a tree classification analysis for the evaluation of the relative importance of each of the possible influential variables considered: gender, age class, education level, professional areas and marital status. The analysis followed the CRT (Classification and Regression Trees) algorithm with cross-validation and with a minimum change in improvement of 0.001, considering a limit of 5 levels and the minimum number of cases for parent or child nodes equal to 25 and 15, respectively.

Factor analysis (FA) was used to assess knowledge based on some data obtained through the items used. Some preliminary assumptions were used to test the suitability of the data before FA, namely the correlation matrix and the values of measure of sample adequacy (MSA), the Kaiser–Meyer–Olkin measure of the adequacy of the sample (KMO) and Bartlett’s test [33]. FA was applied using extraction with principal component analysis (PCA) and Varimax rotation. The number of components retained was established based on the Kaiser criterion, i.e., only eigenvalues ≥ 1 were considered. The communalities allowed assessing the percentage of variance explained (VE) by the factors extracted [34] and a minimum value for this was fixed as 0.4, corresponding to 36% VE [35,36]. The internal consistency in each factor was investigated using Cronbach’s alpha (α) [34,37].

Cluster analysis (CA) was carried out over the FA, using five hierarchical methods (ward, centroid, average linkage within groups, average linkage between groups, complete linkage–furthest neighbour). The coefficients of the agglomeration schedule allowed for the estimation of the most appropriate number of clusters that should be considered. After that, the partitive k-means method was used, considering the five initial solutions obtained using the hierarchical methods previously mentioned, and stability was assessed through contingency tables. Convergence was also evaluated based on cluster centres [38].

The data were processed using the SPSS program, version 27 from IBM, Inc, and the level of significance used was 5%.

### 2.3. Limitations

The sample was recruited by convenience following an invitation sent out via e-mail or social media. This research was initiated in the academic context, a PhD study within the field of medicine, and therefore, because the invitations were sent to personal contacts and personnel in the faculty primarily, it included a high number of young adults, students or recently graduated people, particularly in the health field. Nevertheless, the invitations were also sent to other participants following a snowball approach. As such, it is expected that the distribution of the participants between the different socio-demographic categories might not be even, as what would be the case in a stratified sample. However, despite this limitation, a high number of participants were included in the study, which can shed some light on the consumption of PPB among Portuguese individuals. By including more young people and more educated people in this study, some of the observed trends can be indicative of the consumption patterns of these more represented categories, such as young adults, educated people, and those with studies or work related to health. It is natural that a different sample would lead to slightly different results, and this limitation is important to bear in mind when analysing the results and conclusions of the present work.

## 3. Results and Discussion

### 3.1. Socio-Demographic Characterisation of the Sample

Table 1 depicts the socio-demographic characteristics of the studied individuals. The distribution according to sex was not equal, with more women participating in the survey (71.2%) than men (28.8%). The participants were aged between 18 and 85 years. Most of them were aged between 31 and 50 years (40.5%) or young adults aged between 18 and 30 years (37.5%), with a much lower percentage of senior adults (aged 51 or plus years) (22.0%). The average age of the participants was 38 ± 14 years, slightly lower for women than for men (37 ± 14 and 39 ± 15, respectively).

With respect to marital status, most participants were single (47.7%) or married/living together (45.4%), with only 5.9% divorced/separated and 1.0% widowed. Regarding the highest level of education, most had completed a university degree (83.6%), and the fraction that had only basic schooling was residual (only 10 participants, 1.1%). The great majority are employed (59.8% work for a third party and 5.1% are self-employed) or students (31.0%).

The professional area of the participants (either regarding work or studies) was also assessed, considering its possible influence on the level of information and consumption habits. A high percentage of participants had professional areas related to health (n = 276), but there were also some in the areas of food (n = 76), agriculture (n = 75) and nutrition (n = 25), with 520 participants having studies or work related to other areas.

**Figure 1:**
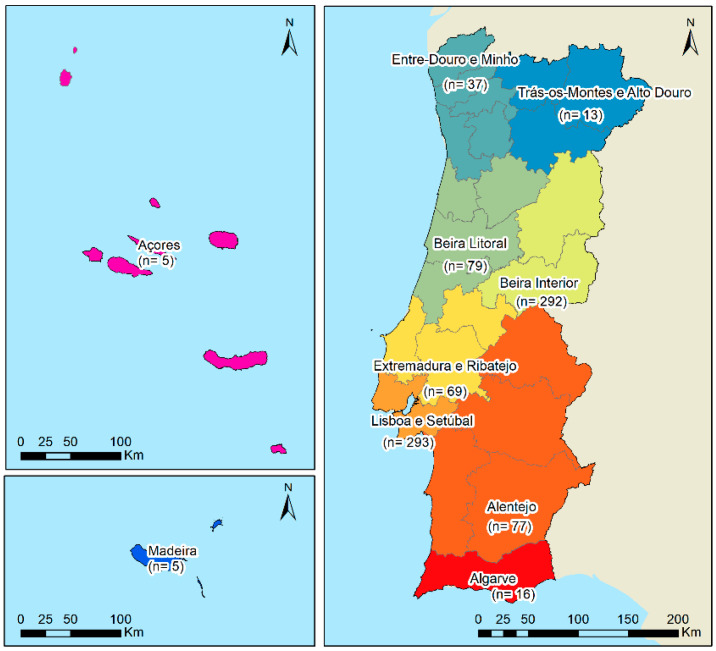
Geographical distribution of the participants.

The participants covered the Portuguese territory, including the archipelagos of Madeira and Azores, although with a more expressive participation from Beira Interior (n = 292) and the metropolitan area of Lisbon and Setúbal (n = 293) (Figure 1).

### 3.2. Consumption Habits Regarding PBB

Slightly more than half of the participants consumed vegetable drinks (54.3%) against 45.7% who did not. Table 2 shows the consumption according to sex and age and reveals significant differences between women and men (*p* < 0.0005), although with a weak association (V = 0.126), but not according to age group (*p* > 0.05).

**Figure 2:**
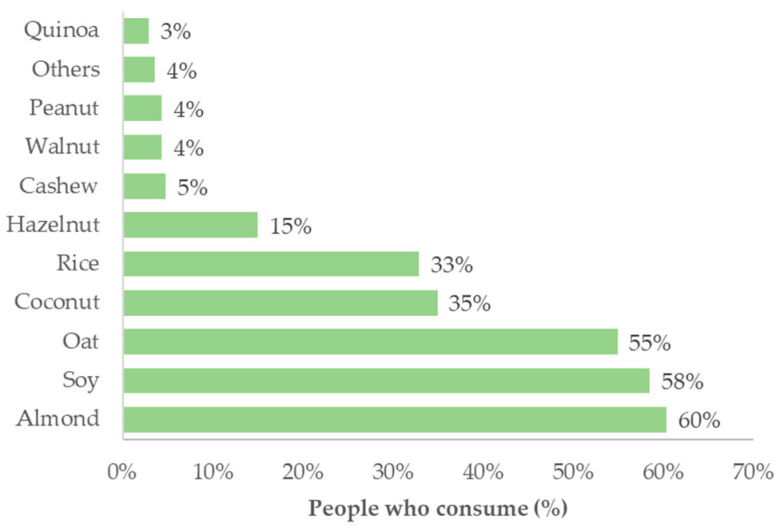
Preferences regarding the type of PBB consumed (N = 481).

Those who consumed this type of product preferred almond, soy and oat beverages, followed by coconut or rice-based beverages. The least preferred products were quinoa, peanut, walnut and cashew-based drinks (Figure 2). In fact, soy beverages contain a higher protein content than other legumes and cereals, and they are the only plant source that contains all nine essential amino acids [39]. Additionally, soy beverages have a high calcium content and are more economically attractive compared to other plant-based alternatives [39]. Nevertheless, the consumption of soy products has been associated with several health benefits [39,40]. These facts could justify why soy-based PBB is the second most consumed by respondents. Soy and almond beverages are among the most commercialised PBB in Portugal, and this availability can also contribute to their broader acceptance and consumption by friends or relatives. The influences from close people who consume certain types of food or beverages are strong motivators for acceptance as well. Different authors have reported the influence of relatives and friends on food intake [41,42].

On the other hand, oat beverages are also a plant-based alternative to dairy, rich in nutrients and low in cost [43]. This plant-based alternative contains a variety of nutrients, including minerals, proteins and fats, as well as other compounds, such as phenols, which are associated with the prevention of various diseases such as diabetes, colon cancer and cardiovascular diseases [44,45,46,47]. Reducing cholesterol and postprandial blood glucose levels are also associated with oat beverages, given their dietary fibre content [43]. These factors could influence consumers’ choice, but in addition to the reported benefits, oat beverages have good organoleptic properties, which may be crucial for the respondents’ choice of this product [43]. Concerning the almond beverage, its flavour and texture dictate its choice by consumers, making this beverage one of the most common in different countries on different continents [48,49]. Similar to the oat beverage, the almond beverage has several nutrients in its composition, such as fibre, proteins and manganese [50]. Additionally, the almond beverages are a source of vitamins, antioxidants and monounsaturated fatty acids, which are considered useful in weight loss [48]. In fact, almond milk has the lowest number of calories, which may dictate the choice of respondents [48].

**Figure 3:**
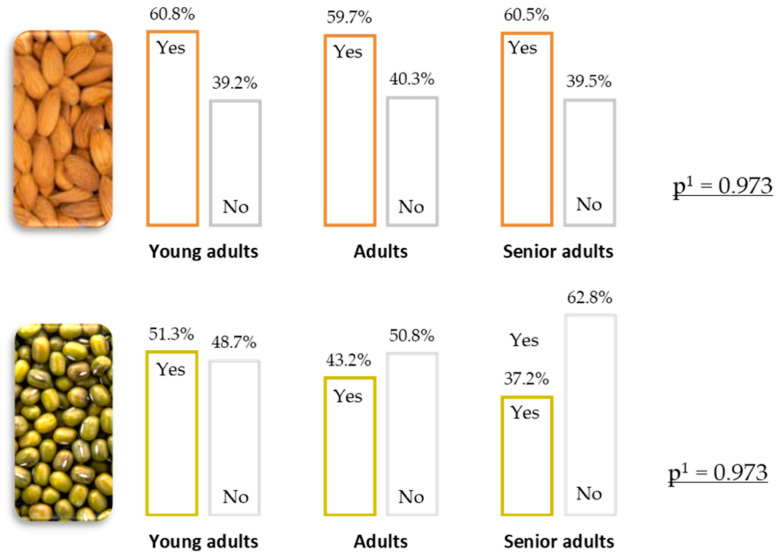
Consumption of some PBB according to age group (^1^ *p*-value of the chi-square test).

For those beverages that were more consumed (almond and soy), it was further investigated if there were statistical differences according to age groups (Figure 3). The result indicated that there were no statistically significant differences in the consumption of any of these two types of beverages (*p* > 0.05), although there is a slight trend for higher consumption among young adults, particularly in the case of soy beverages. These data are in accordance with a study carried out by Wolf et al. [51], which postulates that young people consume less milk than older generations, particularly young people born in the 1990s.

With respect to the frequency of consumption, 37.2% consume PBB rarely (2 to 3 times per month), 30.9% consume PBB sometimes (2 to 3 times per week), 20.0% consume PBB often (once a day) and 11.9% consume PBB more than once per day. Several reasons were pointed out by the participants to consume these products, namely adopting a vegetarian (n = 42) or vegan (n = 18) diet, being intolerant to lactose (n = 101), for health reasons (n = 124), due to nutritional aspects (n = 206), or owing to ethical concerns (n = 112). On the other hand, reasons not to consume include never having tried this type of product (n = 135) although having experimented, did not like it (n = 142), or does not consider this an option more advantageous compared to milk (n = 117).

### 3.3. Acceptability and Motivations

Regarding acceptability, Table 3 shows that the acceptability of products made with PBB was higher when compared to the beverages themselves (62.5% against 41.8% of people with scores higher than 3, respectively).

Concerning the motivations (Table 4) that drive the Portuguese to consume these types of beverages originating from plants, these include the focus on sustainable choices (49.9% of strong motivation), followed by health benefits (46.7% of strong motivation), the diversification of agricultural crops (37.3% of strong motivation) or looking for alternatives to non-dairy products (31.6% of strong motivation). The results further show that the strong and very strong motivations for the health benefits account altogether for 74.1% of the participants, indicating that health concerns are a very important factor driving consumers to these types of products. The desire to follow innovative trends does not seem to influence the participants (55.1% indicated a very low motivation). In fact, the motivations of PBB consumers are complex [52]. However, previous studies revealed that, similarly to what was verified, the main motivations relate to issues of sustainability and animal ethics [53,54]. Another frequently reported strong motivation is related to health and well-being, namely allergy or sensitivity to dairy products, as well as practising vegan or vegetarian diets [55,56,57].

Assuming that the professional area of the participants could be important in determining people’s motivations to consume PBB, the chi-square test was carried out between the motivations and the professional area of the participants. It was observed from Table 5 that, in general, the professional area did not significantly influence people’s motivations for the consumption of PBB, with just a few exceptions. Participants in the area of nutrition showed a stronger motivation to consume PBB because of the perceived health benefits, although the association was weak (V = 0.175) but significant. Regarding this result, it is worth mentioning that the association can be weak, but significant nonetheless, according to the value of *p* that is lower than the significance level considered of 5%. If one association is strong, usually it is also significant, but the other way round is not necessarily true, like in the present case.

Those participants with professional areas or studies related to agriculture showed a stronger motivation to consume these beverages due to their higher sustainability, but again, the association was weak (V = 0.202). Finally, the people with a profession or studies in the domain of health revealed a stronger motivation to consume PBB because these promote the cultivation of a diversity of crops, also with a weak association (V = 0.184).

Although, in general, the professional area does not significantly influence the motivations for consumption of PBB since the values of *p* in Table 5 are mostly over 0.05, there are some significant relations between the professional area and the motivations for the consumption of PBB. Specifically, a significant but weak association was found between “Being a more sustainable choice than milk from animal origin” and a agriculture professional (*p* = 0.002, V = 0.202); a significant but weak association was found between “Promoting the differentiated cultivation of vegetable species” and a health professional (*p* = 0.008, V = 0.184); and finally, a significant but again weak association was found between “Believing they can have benefits for human health” and being a nutritionist (*p* = 0.015, V = 0.175).

Consumers are increasingly interested in making choices that are considered healthier and have health benefits [58]. Some studies suggest the potential of PBBs in reducing the risk of cardiovascular and gastrointestinal diseases, improving the immune system, or decreasing the risk of bone loss [58]. Other beneficial properties, such as antimicrobial and antioxidant, have also been associated with PBBs [58]. Therefore, it is natural that health and nutrition professionals are more motivated to consume these beverages. Additionally, PBB usually undergo a fermentation process, which allows the conversion of complex carbohydrates into simpler molecules, leading to the production of more nutritionally interesting metabolites with bioactive properties [59,60]. However, these products can also be enriched with the incorporation of nutritionally important compounds, improving the quality of PBB and the bioavailability of elements and minerals, improving their interest from a nutritional point of view [59]. In fact, enrichment and the improvement in the health-enhancing properties of PBB is related to motivations for their consumption and this is specifically found with “Believing they can have benefits for human health”, the fifth motivation in Table 5, which is significantly related to the area of nutrition.

Sustainability has become a significant factor for consumers choosing PBB. These alternatives to cow’s milk require considerably less environmental pressure during production [58]. The use of animals makes the milk production process less sustainable; in addition, most of the animals’ feed is used in metabolic processes, increasing the overall metabolic footprint [58,61]. In addition to environmental issues, ethical concerns related to animal welfare have also been associated with dairy production [2,62,63]. Therefore, professionals in the agricultural field naturally have stronger motivations to consume PBB due to its greater sustainability [64]. The motivation “Being a more sustainable choice than milk from animal origin” includes sustainability on a broader concept, i.e., including not only sustainable agricultural practices but also responsible food production and consumption, including animal well-being. This motivation was found significant for professionals in agriculture because they are more alert to the sustainability aspects linked with food production.

### 3.4. Perceptions and Knowledge

The perceptions, knowledge and beliefs regarding PBB were evaluated through a set of 23 statements for which the participant had to indicate the level of agreement on a scale from totally disagree to totally agree. These results are presented in detail in Table A1 in Appendix A. For example, when asked about the lower nutritional value of PBB when compared to milk, people were mostly against this false statement (23.4% totally disagree and 23.6% partially disagree). On the other hand, most participants partially agree (35.6%) or totally agree (37.2%) that PBB are expensive. High agreement was observed for other statements, such as number 11—about the effect in reduction in cholesterol, 16—about being a source of energy, 17—being rich in nutrients, 18—being a source of vitamins, 19—being a source of dietary minerals, or 20—containing bioactive compounds.

#### 3.4.1. Factor Analysis

The 23 statements were included in the FA, but several trials indicated that some of the items would not be suitable. Hence, the final items included were 8, 9, 11, 13, 14, 15, 16, 17, 18, 19, 21, 22 and 23. For this group, Bartlett’s test indicated the adequacy of the data to apply FA with a *p*-value highly significant (*p* < 0.0005), leading to the rejection of the null hypothesis H0: The correlation matrix is equal to the identity matrix. The correlation matrix showed that all values were higher than the minimum threshold acceptable of 0.5 (varying between 0.753 for item 15 and 0.910 for item 21). The value of KMO was 0.853, which is considered good, according to the classification of Kaiser and Rice [65], and also indicates the suitability of the data for the application of FA. The solution was obtained after Varimax rotation in five iterations and explained 57.3% of the total variance. It retained four components (factors), with percentages of total variance explained as follows: F1—22.4%, F2—21.27% and F3—13.8%. Item 18 had the largest fraction of VE by the solution, 81.5%, followed by item 14 with 80.3% VE. None of the variables had communalities lower than 0.4, so this solution was considered final and is presented in Table 6.

The first factor (HB) was identified as relating to the health benefits of the PBB, with higher loadings for reduction in body fat (0.746) and anti-inflammatory properties (0.690), meaning that these two items contributed in a higher degree to the definition of the factor. Factor two (EN) was identified as linked with the energetic value and nutrition, and the two variables with higher loadings were related to the richness in vitamins and minerals (0.815 and 0.783, respectively). The third factor was interpreted as being associated with traditional consumption of these beverages (CT) and contained three items, with loadings variable between 0.635 and 0.803. Because all the items had loadings higher than 0.4, this solution is acceptable with all the ten variables included [35].

The Cronbach’s alpha (α), was used to validate the internal consistency within each of the factors [34]. The values of Cronbach’s alpha for factors F1 (HB) and F2 (EN) were higher than 0.8, thus being considered good [65,66,67]. However, the value of alpha for factor F3 (CT) was lower, but still, according to some authors, could be acceptable [65,66,67], even though the desirable value should be equal to 0.7 or higher [65,66].

#### 3.4.2. Cluster Analysis

The scores that resulted from FA were used for the CA. The starting phase used CA by applying five different hierarchical methods to establish the number of clusters, which in the present case was three, based on the coefficients of the agglomeration schedule. For this number of clusters, the solutions obtained with the different hierarchical methods were checked for compatibility through contingency tables, showing the percentage of cases allocated to the same clusters as varying from a minimum of 44% to a maximum of 80% (Table A2 in Appendix B). Based on these results, the two highest compatibilities were found for ward *versus* average linkage within groups (80%) and centroid versus average linkage between groups (77%). Hence, these were the four solutions recommended for use as the initial solution for the k-means clustering analysis. The results presented in Table 7 for the cluster centres and the number of cases resulting from the k-mean with the different initial solutions reveal a convergence to very similar solutions and in two cases the exact same solution (initial solutions centroid and average linkage within groups), thus ensuring stability. Hence, this was considered the final solution. ANOVA indicated high values of the statistic for factors F1 and F2 (Fstatistic = 243.9 for F1 and Fstatistic = 282.5 for F2, with *p* < 0.001 in both cases) and a little lower for F3 (Fstatistic = 38.0, with *p* < 0.001). These results confirm the similarity between the cases within each of the factors and the differences between factors. Finally, based on the values of Fstatistic, which are of the same order of magnitude for F1 and F2 but lower for F3, it is concluded that F3 contributes differently than F1 and F2 for group discrimination.

Based on the results in Table 7, the clusters can be interpreted as follows:

- Cluster C1: negative input in all factors;
- Cluster C2: negative input for F1 (Health Benefits) and F3 (Culture and Tradition), but positive for F2 (Energy and Nutrition);
- Cluster C3: positive input in all factors.

The explanation of the clusters is the following: Cluster C1 includes people that do not value any of the issues considered in the factors, namely Health Benefits, Energy and Nutrition or Culture and Tradition; Cluster C2 includes individuals that only value the aspects linked with Energy and Nutrition, i.e., they give attention to these aspects; finally, Cluster C3 includes the participants that give attention to all the aspects considered, i.e., Health Benefits, Energy and Nutrition and Culture and Tradition.

#### 3.4.3. Tree Classification of the Level of Knowledge

The answers to the statements used to assess the knowledge were used to calculate an average value for each participant, measuring the level of knowledge on a scale from 0 to 5 points: very weak knowledge—score ∈ [0;2], weak knowledge—score ∈ [2;3], good knowledge—score ∈ [3;4] and very good knowledge—score ∈ [4;5]. In order to calculate this average score, some false statements were reversed, thus following a common trend. The results revealed that the average values of knowledge for each participant varied from a minimum of 0 to a maximum of 4.35, with a mean of 2.77 and a standard deviation of 0.79. The distribution of the participants by classes of knowledge was 16.3% with very low knowledge, 35.4% with low knowledge, 47.3% with good knowledge and 1.0% with very good knowledge. This knowledge was not significantly variable with regard to sex (chi-square test *p* = 0.201), age class (chi-square test *p* = 0.500) or professional status (chi-square test *p* = 0.715).

**Figure 4:**
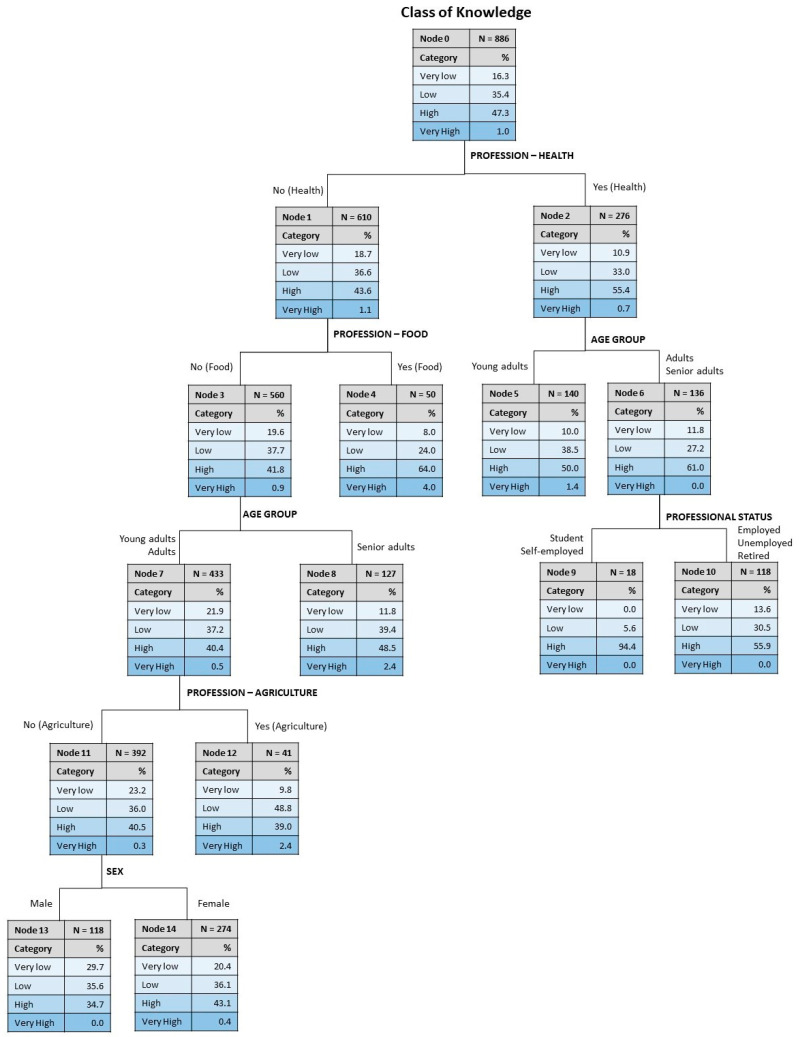
Tree classification for the level of knowledge about PBB.

A tree classification was conducted to understand the relative influence of the socio-demographic variables on the level of knowledge, the results being shown in Figure 4. For this, the variables included in the analysis were sex, education, professional status, marital status, age group, profession—agriculture, profession—health, profession—nutrition and profession—food, but only six of them were found explicative. The resulting tree had five levels of depth, fifteen nodes, of which eight were terminal, and allowed an overall percentage of correct classification equal to 47.9%. The risk estimate for resubstitution was 0.521, with a standard error of 0.017, and the risk for cross-validation was 0.533, with a standard error of 0.017.

The results in Figure 4 show that from the variables included in the analysis, the profession related to health was the most important predictor for the level of knowledge, with those whose professional activity was related to health showing higher percentages of participants in the class of high knowledge (55.4%). For the health professionals, the following discriminating variable was age, separating the young adults with less incidence of high knowledge (50.0% against 61.0%). The third discriminating variable for the health professionals aged over 30 years was professional status, with students and the self-employed revealing a higher proportion in the category of high knowledge (94.4%). As for the other branch of the tree, i.e., those whose professional activity was not related to health, the following discriminating variable was professional activity in the area of food, with these professionals revealing higher knowledge (64.0% in the high knowledge category), this being a terminal node. For the food professionals, age was the next discriminant, with lower knowledge for the group aged under 50 years. The following discriminant was a profession related to agriculture, and the last was sex, with women showing a higher percentage in the class of high knowledge (43.1%).

Overall, these results seem to indicate that the most relevant discriminating factors for knowledge about PBB were health profession, age, professional status, food profession, agriculture profession and gender. On the other hand, education, marital status and profession in nutrition do not significantly impact knowledge about PBB.

In fact, the potential health benefits associated with the consumption of PBB [58], as well as the fact that they constitute an alternative to dairy products for patients with allergies and intolerances [4], could justify that profession—health was found to be a factor that determines knowledge about PBB. The fact that consumers demand a constant organoleptic improvement of PBB and their concerns about sustainability could also justify the reason for the profession—food and profession—agriculture factors [58].

## 4. Conclusions

This study provided a deeper understanding of the consumption habits, perceptions and knowledge of PBB in Portugal. More than half of the respondents consumed PBB, with the majority being adults aged 31 to 50 years and a higher percentage of women. Among the PBB options, almond, soy and oat beverages were the most consumed, followed by coconut and rice-based beverages. In contrast, quinoa, peanut and cashew beverages were the least preferred.

The largest fraction of PBB consumers consume PBB between 2 and 3 times a month, but a close percentage consume PBB 2 to 3 times a week. A smaller percentage consume PBB once a day, and another smaller percentage even more than once a day. Pertaining to the reasons for consuming PBB, it was found that the majority do so for nutritional reasons, followed by health reasons and ethical reasons and a smaller percentage due to practising a vegetarian or vegan diet. There was also a focus on sustainable choices, the diversification of agricultural product crops and, finally, the search for alternatives to non-dairy products.

Besides understanding consumer habits, this work also made it possible to assess the respondents’ knowledge of PBB. Having a health-related profession was the most important predictor of the knowledge level. Within this group, there were discriminating variables of age, with young adults showing a lower level of knowledge and professional status, with students and independent workers revealing greater knowledge. Factors such as education, marital status, and profession in the area of nutrition did not present a discriminating capacity regarding knowledge about PBB.

As stated previously, this work has some limitations, mainly regarding the samples used for the study, because it was a convenience sample with uneven distribution among the considered socio-demographic categories. Specifically, there were fewer men (~30%) than women, fewer older people (~22% aged 51 or plus) and fewer divorced/separated/widowed participants (~7%), contrasting with high rates of participants with a university degree (~84%), a high number of employed people (~60%) and professionals related with health (~31%). As such, the conclusions obtained must be seen in the context of the used sample, and this is particularly relevant when it comes to issues such as consumption trends and the level of information. In fact, younger people tend to follow more recent food consumption trends, and the use of PBB is a relatively recent expanding trend to find vegetable-based alternatives to animal milk. On the other hand, the level of information tends to be higher in people with a higher level of studies, and in what concerns health benefits, the knowledge tends to be higher in professionals linked with health.

## Figures and Tables

**Table 1:** Socio-demographic characterisation of the Portuguese sample (N = 886).

Variable	Class	N	%
Sex	Female	631	71.2
	Male	255	28.8
Age group	Young adults (18–30 y)	339	37.5
	Adults (31–50 y)	366	40.5
	Senior adults (≥51 y)	181	22.0
Marital status	Single	423	47.7
	Married or living together	402	45.4
	Divorced or separated	52	5.9
	Widowed	9	1.0
Education level	Basic School	10	1.1
	Secondary School	135	15.3
	University Degree	741	83.6
Professional status	Student	275	31.0
	Self-employed	45	5.1
	Employed	530	59.8
	Unemployed	26	3.0
	Retired	10	1.1

**Table 2:** Consumption of PBB according to sex and age group.

	Sex		Age		
	Female	Male	Young Adults	Adults	Senior Adults
Consume	58.3%	44.3%	55.8%	56.3%	47.5%
Do not consume	41.7%	55.7%	44.2%	43.7%	52.5%
*p*	<0.0005 ^1^		0.121 ^2^		

^1^ *p*-value of Fisher’s exact test; ^2^ chi-square test.

**Table 3:** Acceptability of PBB or derived food products.

	Acceptability Levels ^1^				
	1	2	3	4	5
Acceptability/predisposition to consume beverages originating from plants	2.2%	15.8%	39.8%	29.4%	12.8%
Acceptability towards consumption of dishes confectioned with PBB	1.2%	8.4%	27.9%	38.8%	23.7%

^1^ Scale from 1 = definitely would not consume to 5 = definitely would consume.

**Table 4:** Motivations for the consumption of PBB or derived food products.

Reasons	Motivation Intensity (% of Participants)				
	Very Weak	Weak	Indifferent	Strong	Very Strong
Being a more sustainable choice than milk from animal origin	2.5	12.3	15.6	49.9	19.8
Wanting to try alternative products to milk	14.6	19.8	26.7	31.6	7.4
Promoting the differentiated cultivation of vegetable species	8.4	12.6	34.8	37.3	6.9
Wanting to follow innovative trends	55.1	15.1	24.7	4.0	1.2
Believing they can have benefits for human health	4.4	8.9	12.6	46.7	27.4
Increasing the area cultivated with these types of crops	12.8	15.8	38.5	24.9	7.9

**Table 5:** Associations between professional areas and the motivations for the consumption of PBB or derived food products.

Reasons to Consume PBB	Chi-Square Test ^1^			
	Nutrition	Food	Agriculture	Health
Being a more sustainable choice than milk from animal origin	0.781	0.766	0.002 (0.202)	0.100
Wanting to try alternative products to milk	0.652	0.868	0.278	0.313
Promoting the differentiated cultivation of vegetable species	0.310	0.624	0.630	0.008 (0.184)
Wanting to follow innovative trends	0.797	0.767	0.560	0.201
Believing they can have benefits for human health	0.015 (0.175)	0.811	0.189	0.989
Increasing the area cultivated with these types of crops	0.431	0.903	0.698	0.865

^1^ *p*-value (Cramer’s coefficient, V, if the value of *p* was significant).

**Table 6:** Results of the factor analysis.

Factor	Item	Loading	Cronbach’s Alpha
F1 (HB: Health Benefits)	8	0.746	0.807
	9	0.632	
	11	0.614	
	21	0.596	
	22	0.647	
	23	0.690	
F2 (EN: Energy and Nutrition)	16	0.701	0.805
	17	0.686	
	18	0.815	
	19	0.783	
F3 (CT: Culture and Tradition)	13	0.635	0.694
	14	0.803	
	15	0.789	

**Table 7:** Results of the cluster analysis.

Initial Solution	Factor		Cluster Centres	
		C1	C2	C3
Ward		(n = 221)	(n = 232)	(n = 433)
	F1	−0.367	−1.003	0.725
	F2	−1.337	0.803	0.252
	F3	−0.339	−0.188	0.274
Centroid		(n = 228)	(n = 227)	(n = 431)
	F1	−0.375	−1.003	0.727
	F2	−1.296	0.827	0.250
	F3	−0.382	−0.168	0.291
Average Linkage Within Groups		(n = 228)	(n = 227)	(n = 431)
	F1	−0.375	−1.003	0.727
	F2	−1.296	0.827	0.250
	F3	−0.382	−0.168	0.291
Average Linkage Between Groups		(n = 228)	(n = 229)	(n = 429)
	F1	−0.375	−0.996	0.731
	F2	−1.296	0.827	0.247
	F3	−0.382	−0.1680	0.288

## Data Availability

The original contributions presented in the study are included in the article, further inquiries can be directed to the corresponding authors.

## Appendix A

Table A1 shows the percentage of answers to the 23 statements used to assess the perceptions, knowledge and beliefs of the participants towards PBB.

**Table A1:** Perceptions, knowledge and beliefs about the PBB.

Perceptions, Knowledge and Beliefs	Don’t Know	Totally Disagree	Partially Disagree	Neither Agree or Disagree	Partially Agree	Totally Agree
1. PBB have less nutritional value than milk	11.5	23.4	23.6	20.1	15.8	5.6
2. PBB are difficult to digest	11.5	40.0	22.6	13.8	6.0	6.0
3. PBB have a greater risk of pesticide residues than milk and dairy	17.2	23.7	21.3	21.0	13.9	2.9
4. PBB are expensive	7.1	2.1	7.8	10.2	35.6	37.2
5. PBB have an unpleasant colour and taste	8.1	27.4	20.2	17.2	19.0	8.1
6. There are a wide variety of PBB on the market	11.3	3.2	7.6	11.9	25.2	41.0
7. The consumption of PBB helps alleviate the symptoms of menopause	53.3	3.3	4.4	30.6	5.8	2.7
8. Replacing milk with PBB contributes to the reduction in body fat	27.5	5.8	11.2	25.6	18.7	11.2
9. The consumption of PBB helps to strengthen bones	30.9	7.9	14.2	28.0	11.6	7.3
10. Herbal drinks have a lower risk of antibiotics than milk and milk products	29.7	6.2	7.6	20.8	19.1	16.7
11. The consumption of PBB helps to reduce cholesterol	25.7	3.4	6.1	20.7	27.3	16.8
12. Vegetable drinks are an option for lactose intolerants	12.2	3.4	2.6	5.5	18.5	57.8
13. In developed countries PBB are not consumed	16.5	52.6	14.4	10.9	3.5	2.0
14. PBB are part of western culture	25.6	15.8	16.9	26.3	10.6	4.7
15. PBB are characteristic of developed counties	20.3	10.0	12.6	21.2	22.9	12.9
16. PBB are a source of energy	14.9	2.8	6.5	21.0	32.1	22.7
17. PBB are rich in macro and micronutrients	24.9	2.0	7.0	15.1	30.0	20.9
18. PBB contain vitamins	13.8	2.7	4.7	12.2	36.0	30.6
19. PBB contain dietary minerals, such as calcium, iron and magnesium	18.3	2.9	6.8	15.3	29.6	27.1
20. PBB contain bioactive compound with health benefits	25.2	2.8	4.3	16.4	28.1	23.3
21. PBB are a source of vitamin D	34.1	4.6	7.7	20.4	18.7	14.4
22. PBB have antioxidant properties	32.7	3.5	7.0	19.6	21.6	15.6
23. PBB have anti-inflammatory properties	37.6	3.5	6.9	21.0	18.5	12.5

## Appendix B

Table A2 shows the comparison of the solutions obtained with the five hierarchical methods tested, showing the percentage of common classification of the cases into the clusters as given by the contingency tables.

**Table A2:** Comparison of the solutions obtained with the hierarchical methods.

Method ^1^	AL-BG	AL-WG	CL-FN	CENT	WARD
AL-BG	—	—	—	—	—
AL-WG	70%	—	—	—	—
CL-FN	59%	70%	—	—	—
CENT	77%	49%	44%	—	—
WARD	69%	80%	57%	57%	—

^1^ AL-BG: average linkage between groups; AL-WG: average linkage within groups; CL-FN: complete linkage–furthest neighbour; CENT: centroid.
